# Genetic variants of *SLC6A4* and risk of coronary artery disease: insights from North Indian population

**DOI:** 10.1186/s13023-025-03761-w

**Published:** 2025-05-14

**Authors:** Jyotdeep Kour Raina, Minakashee Sharma, Ravi Sharma, Rohit Bhardwaj, Parvinder Kumar, Santasree Banerjee, Rakesh Kumar Panjaliya

**Affiliations:** 1 Department of Human Genetics, Government Gandhi Memorial Science College, Cluster University of Jammu, Jammu & Kashmir, Jammu, 180016 India; 2 Institute of Human Genetics, University of Jammu, Jammu & Kashmir, Jammu, 180016 India; 3 Department of Zoology, University of Jammu, Jammu & Kashmir, Jammu, 180016 India; 4 Department of Zoology, Government Gandhi Memorial Science College, Cluster University of Jammu, Jammu & Kashmir, Jammu, 180016 India; 5 Department of Genetics, College of Basic Medical Sciences, Jilin University, No.126 Xinmin Street, Changchun, 130021 Jilin China

**Keywords:** Coronary artery disease, Cardiovascular disease, *SLC6A4* gene, 5-HTT, 5-HTTLPR, rs25531, STin2, Polymorphism

## Abstract

**Background:**

The activity of *SLC6A4* is influenced by its polymorphisms, including the length variation in serotonin transporter linked promoter region (5-HTTLPR), a single nucleotide polymorphism (rs25531), and variable number of tandem repeats in serotonin transporter intronic enhancer (STin2). These polymorphisms have been implicated in the development of vascular diseases. Our research aimed to determine whether the bi-allelic 5-HTTLPR, tri-allelic 5-HTTLPR (rs25531), and STin2 polymorphisms of *SLC6A4* were associated with an increased risk of coronary artery disease (CAD) in the North Indian population of Jammu region in Jammu and Kashmir state of India.

**Methods:**

In this study, we performed a large cohort case-control study. Here, we recruited 400 patients clinically diagnosed with CAD, and 400 unrelated healthy individuals with similar sex and age range. We performed Polymerase Chain Reaction (PCR) for genotyping the 5-HTTLPR and STin2 polymorphisms. In addition, PCR- Restriction Fragment Length polymorphism (RFLP) was used to perform restriction fragment length polymorphism for the rs25531. Finally, we performed statistical analysis with the yield data.

**Results:**

The L-allele of 5-HTTLPR was significantly associated with CAD susceptibility, with an odd ratio (OR) of 1.39 and a *p*-value of 0.01. However, no significant association was identified for the tri-allelic 5-HTTLPR (rs25531) and STin2 polymorphism with the susceptibility of CAD. The haplotype combinations associated with CAD outcomes include L-12 and LA-10.

**Conclusions:**

Although, majority of the previous studies have evaluated the association of 5-HTTLPR biallelic polymorphism with CAD, our findings suggested that the tri-allelic 5-HTTLPR (rs25531) is a more reliable candidate than the bi-allelic 5-HTTLPR, as studying the bi-allelic version alone may generate association bias. Based on the results of this study, the rs25531 and STin2 polymorphisms indicated that the *SLC6A4* gene does not contribute to the development of CAD in the population of the of Jammu region in Jammu and Kashmir state of India.

## Background

Coronary artery disease (CAD) is the leading global cause of mortality [1]. Common variant association studies have identified approximately 60 genetic loci with increased risk of CAD [2]. CAD is a chronic form of cardiovascular disease (CVD) which affects blood vessels for supplying blood to cardiac muscles [3]. Recently, the incidence of CAD is exponentially increasing in the population of Indian subcontinent, and it has become a great challenge for healthcare systems to curb its prevalence [4]. CAD has a multi-factorial etiology, with many interlinked risk factors such as ageing, varying lifestyles, food habits, psychological facts and genetic background [4].

**Fig. 1:**
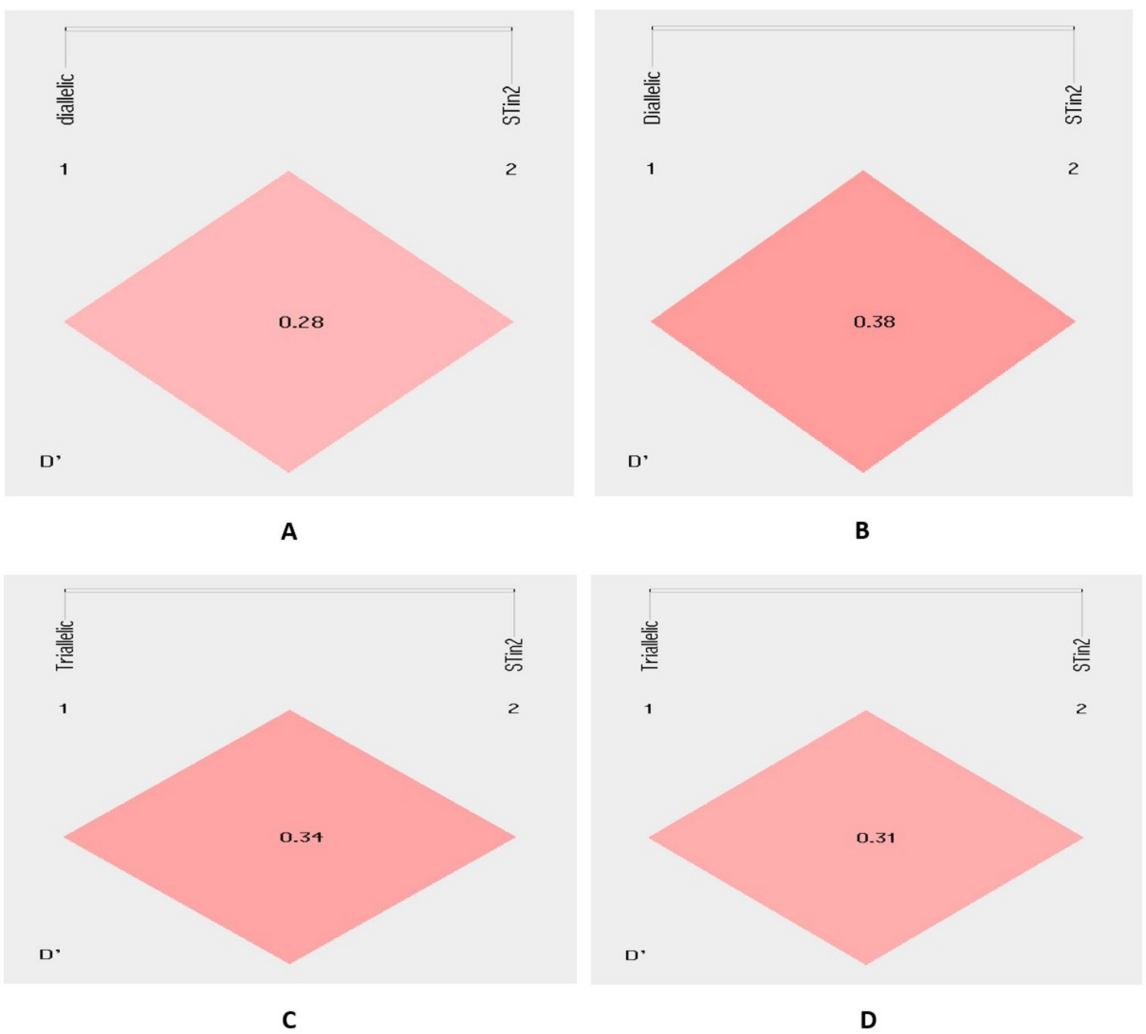
Linkage Disequilibrium (LD) plot for SLC6A4 gene polymorphisms (**A** & **C**) CAD cases (**B** & **D**) Controls (The numbers inside every box represent D’ values (%) of the LD)

Serotonin (5-hydroxytryptamine, 5-HT) is a potent neurotransmitter primarily synthesized in the brain [5]. However, the production of serotonin is also occurring locally in the heart, kidney and adrenal gland, whereas platelets are well known to store but not synthesize serotonin. The functional activity of serotonin is governed by the specialized transporters (SERT or 5-HTT), which are synthesized by the *SLC6A4* (also named as 5-HTT) gene [6]. These transporters are engaged in the reuptake of serotonin from the extracellular spaces and, maintain the duration and strength of the interactions between serotonin and its receptors [7]. The SERT transporters are also active in cardiovascular physiology and are significantly involved in processes like modulation of cardiac and smooth muscle contractility, platelet aggregation and cellular mitogenesis [8]. The human serotonin transporter gene is confined to the genomic location at 17q11.2, spans 31 kb, contains 14 exons and has several polymorphisms associated with differential expression of serotonin transporter [9] (Fig. 1).

A variable number tandem repeat (VNTR) polymorphism (GC-rich repetitive sequence) is located in the promoter region of the *SLC6A4*, nearly 1 kb upstream of the transcription initiation site with an insertion/deletion (Indel) variation of 44 bp, creating a Long (L) and a Short (S) allele, respectively [10, 11]. The L allele has 16 repeats (each 20–23 bp long), while the S allele has 14 repeats [12]. This bi-allelic polymorphism is named 5-HTTLPR (5-Hydroxytryptamine Transporter-Linked Polymorphic Region) polymorphism. The S-allele of HTTLPR is responsible for decreased transcriptional activity (less 5-HTT mRNA transcript) and decreased 5-HT uptake, resulting in a longer duration of serotonergic activity [13, 14]. In contrast, L-allele is associated with approximately three times higher basal activity [15]. Additionally, the activity of the 5-HTTLPR alleles is modified by an SNP, i.e. A to G transition (rs25531) within the region (5-HTTLPR), which modifies the activity of both L and S alleles [16, 17]. It has been demonstrated by Hu et al. that LA haplotype is associated with approximately twice the amount of mRNA expression compared to SA, SG and LG [18]. Based on transcriptional activity, all 3 variants viz. SA, SG and LG are designated as S’, thus resulting in the tri-allelic classification of 5-HTTLPR where LALA is highly expressing, LAS’ is with intermediate expression, and S’S’ is of low expressing genotype [19]. The third variation in the SLC6A4 gene is Serotonin Transporter Intronic VNTR Enhancer (STin2) polymorphism, which is a functional 17 bp VNTR polymorphism in the second intron (rs57098334) and has three alleles: 9, 10 and 12 repeats [20]. A high expression of SERT has been correlated with an increasing number of STin2 repeats.

It is postulated that the presence of L-allele can contribute to undesirable cardiovascular phenotypes such as myocardial infarction, pulmonary hypertension and can be the front runner in evaluating the genetic predisposition for the development of CAD. However, the association of the development of CAD with 5-HTTLPR (rs25531) and STin2 has not been exploited much. Although, there are reports on the potential engagement of these *SLC6A4* gene variants with psychiatric conditions worldwide [15, 18, 21], the variants have not been explored to a great extent, especially about their involvement in risk of developing CAD phenotype. We performed the case-control study to evaluate the association of *SLC6A4* gene variants with genetic predisposition to CAD in the North Indian population of Jammu and Kashmir.

## Methods

### Study subjects

The present study includes 400 clinically confirmed cases of CAD and 400 unrelated healthy controls were belonging to different areas of the Jammu region of the Union Territory of Jammu and Kashmir (India). The cases were enrolled from the Out Patient Department of Cardiology, ASCOMS, Sidhara, Jammu and private clinics. The healthy (blood donors, hospital staff and volunteers) controls were enrolled in the present study without a prior history of CVD or other chronic disease.

### Inclusion and exclusion criteria

All the patients had significant coronary artery disease. Clinical diagnosis has confirmed by coronary angiography with more than 50% stenosis in at least one epicardial coronary artery. Patients with isolated hypertension, heart valve problems, arrhythmia, stroke and congestive heart failure were excluded. Enrolled controls did not have any form of cardiovascular diseases (CVD). Individuals having abnormal glucose tolerance, dyslipidemia, and consuming anti-hypertensive medications were not considered as controls.

### Ethical authorization

The concerned Ethical Committee, University of Jammu, gave ethical approval for conducting the present study. Each study participant was made aware of the nature and scope of the study. An informed written consent was duly obtained from all the study participants.

### DNA isolation and genotyping

Whole blood was collected in 2 ml of EDTA-coated vials and stored at -20°C prior to DNA extraction. Genomic DNA was extracted from collected blood by the phenol-chloroform method [22]. Genotyping for the 5-HTTLPR VNTR was performed by polymerase chain reaction (PCR) using forward primer: 5’-TCCTCCGCTTTGGCGCCTCTTCC-3’ and reverse primer: 5’-TGGGGGTTGCAGGGGAGATCCTG- 3’. Amplification was performed in a final volume of 25 µl containing 2 µl genomic DNA, 5 µl flexi buffer (5X), 2.5 µl MgCl2 (25mM), 0.5 µl dNTPs (10 Mm), 0.2 µl Taq (5U/µl), 0.5 µl each primer (100pmol/µl) and 13.8 µl PCR water to make up the final volume. Thermocycler (Veriti, Applied Biosystems) conditions were as follows: Pre-denaturation at 95 °C for 15 min, followed by 35 cycles of denaturation at 94 °C for 30 s, annealing at 65.5 °C for 90 s and initial extension at 72 °C for 1 min with a final extension at 72 °C for 10 min. For genotyping of rs25531 SNP, the amplicons were given overnight digestion with HpaII (New England Biolabs) restriction enzyme at a temperature of 37 °C. The products were analysed on 4.5% agarose gel. The primer sequence for the STin2 locus was forward primer: 5’-GGGCAATGTCTGGCGCTTCCCCTACATA-3’ and reverse primer: 5’-TTCTGGCCTCTCAAGAGGACCTACAGC-3’. The PCR conditions were the same as described above. The band sizes for PCR and RFLP products are mentioned in Table 1.

### Statistical analysis

Genotypic and allelic frequencies were calculated by the direct counting method. Hardy-Weinberg Equilibrium (HWE) and the differences in genotypic frequencies were examined by using the Chi-square test. To assess the association of increased risk of CAD with SLC6A4 gene variants, odds ratio (OR) with 95% confidence interval (CI) were calculated under different genetic models adjusted for age, sex, body mass index (BMI) and Waist hip ratio (WHR) by Statistical Package for Social Sciences (SPSS) version 20 software. A value of *P* < 0.05 was considered as statistically significant. SHEsis software was used to calculate the haplotype frequencies and pairwise linkage disequilibrium (LD) and its respective measures (D’ & r2) separately for both 5-HTTLPR-STin2 and rs25531-STin2 variants among cases and controls [23].

## Results

### Association of *SLC6A4* gene polymorphisms with increased risk of CAD 

Genotypic and allelic distribution of three *SLC6A4* gene polymorphisms (bi-allelic 5-HTTLPR, tri-allelic 5-HTTLPR (rs25531) and STin2) among CAD patients and controls are summarized in Table 2. There was no deviation from the Hardy-Weinberg Equation in allele frequencies.

**Table 1:** Band size of PCR and RFLP products of SLC6A4 gene variants

S.No.	Gene Variant	Band Size (bp)
1.	*VNTR 5-HTTLPR* L-allele S-allele	512 bp (PCR) 469 bp (PCR)
2.	*SNP rs25531(FN)* S_A_ S_G_ L_A_ L_G_	469 bp (RFLP) 402, 67 bp (RFLP) 512 bp (RFLP) 402, 110 bp (RFLP)
3.	*VNTR STin2* 12- repeats 10-repeats 9-repeats	300 bp (PCR) 267 bp (PCR) 250 bp (PCR)

* S_**A**_, **S**_**G**_**and L**_**G**_**were taken as S**_**+**_

**Table 2:** Genotype distribution and allele frequencies of *SLC6A4* gene polymorphisms among study groups

POLYMORPHISM/ GENOTYPE/ ALLELE	CAD PATIENTS (*n* = 400) (%)	CONTROLS (*n* = 400) (%)	OR (95% CI)	Adjusted *p*-value
***5-HTTLPR*** SS SL LL S L χ^2^	210 (52.5%) 150 (37.5%) 40 (10%) 0.7 0.3 2.86 (*p* = 0.1)	245 (61.25%) 130 (32.5%) 25 (6.25%) 0.77 0.23 1.85 (*p* = 0.2)	1 (Ref.) 1.35 (1.0-1.81) 1.87 (1.10–3.18) 1 (Ref.) 1.39 (1.11–1.74)	0.05 0.03* 0.01*
***rs25531*** S’ S’ S’L_A_ L_A_L_A_ S’ L_A_ χ^2^	276 (69%) 107 (26.75%) 17 (4.25%) 0.82 0.18 2.48 (*p* = 0.11)	288 (72%) 97 (24.25%) 15 (3.75%) 0.84 0.16 3.39(*p* = 0.06)	1 (Ref.) 1.15 (0.83–1.59) 1.18 (0.58–2.41) 1(Ref.) 1.13 (0.87–1.47)	0.6 0.64 0.9
***VNTR STin2*** 12/12 rpts 12/10 rpts 10/10 rpts 12 rpt 10 rpt **χ** ^**2**^	190 (47.5%) 171 (42.75%) 39 (9.75%) 0.69 0.31 0.50 (*p* = 0.47)	180 (45%) 167 (41.75%) 53 (13.25%) 0.66 0.34 2.04 (*p* = 0.15)	1 (Ref.) 0.97 (0.72–1.03) 0.70 (0.44–1.11) 1 (Ref.) 0.87 (0.71–1.07)	0.8 0.3 0.3

*Significant *p*-values

The frequencies of the L allele and LL genotype of bi-allelic 5-HTTLPR polymorphism were significantly higher in CAD patients than in controls [L vs. S: OR = 1.39 (1.11–1.74), *p* = 0.001 and LL vs. SS: 1.87 (1.10–3.18), *p* = 0.03]. However, when participants were stratified by tri-allelic 5-HTTLPR genotypes, we did not observe a statistically significant difference in the high expressing LA allele and LALA genotype distribution among CAD patients and controls [LA vs. S’: OR = 1.13 (0.87–1.47), *p* = 0.9 and LALA vs. S’S’: OR = 1.18 (0.58–2.41), *p* = 0.64]. Furthermore, it was noticed that 10/10 vs. 12/12 repeats for STin2 genotypes do not have any significant difference between CAD patients and controls.

The *SLC6A4* gene polymorphisms were assessed for possible CAD association, and ORs at 95% CI were calculated for dominant and recessive models adjusted for age, sex and BMI (Table 3). The dominant model analysis found that SL + LL genotypes of bi-allelic 5-HTTLPR conferred 1.4-fold risk (*p* = 0.02). In contrast, none of the applied genetic models depicted a significant association between tri-allelic 5-HTTLPR (rs25531) polymorphism and susceptibility to CAD. Similarly, we have not observed a significant association of STin2 polymorphism with the increased risk of CAD.

**Table 3:** Association between *SLC6A4* gene polymorphisms with CAD

MODEL	CAD PATIENTS (*N* = 400)	CONTROLS (*N* = 400)	OR (95% CI)	Adjusted *p*- value
*5-HTTLPR* **Dominant** SL + LL vs. SS **Recessive** LL vs. SL + SS	190/210 40/360	155/245 25/375	1.43 (1.08–1.89) 1.67 (0.99–2.80)	0.02* 0.05
*rs25531* **Dominant** S’L_A_+L_A_L_A_ vs. S’S’ **Recessive** L_A_ vs. S’L_A_+ S’S’	124/276 17/383	112/288 15/385	1.16 (0.85–1.57) 1.14 (0.56–2.31)	0.6 0.7
*VNTR STin2* **Dominant** 12/10 + 10/10 vs. 12/12 **Recessive** 10/10 vs. 12/10 + 12/12	210/190 39/361	220/180 53/347	0.90 (0.68–1.19) 0.71 (0.46–1.10)	0.5 0.2

*Significant *p*-values

Separate comparison of haplotype frequencies of bi-allelic 5-HTTLPR -STin2 and triallelic 5-HTTLPR (rs25531)-STin2 variants among CAD patients and controls are given in Tables 4 and Table 5 respectively. The haplotype L-12 provided 1.45-fold risk of development of CAD whereas haplotypes S-10 and S’-10 were attributing about 1.3 (1/0.29) and 1.4 (1/0.25) fold significant protection against CAD respectively. Moreover, estimating the degree of LD, LD coefficients and haplotype block structure were obtained based on measures of LD values for both bi-allelic 5-HTTLPR-STin2 and tri-allelic 5-HTTLPR (rs25531)-STin2 polymorphisms (Fig. 1). According to the measures of linkage disequilibrium (LD), it was inferred that the bi-allelic 5-HTTLPR-STin2 and tri-allelic 5-HTTLPR (rs25531)-STin2 variants were in slight LD among both CAD patients and controls. However, for bi-allelic 5-HTTLPR -STin2, the values observed for D’ and r2 in CAD patients were 0.28 and 0.07 respectively and in controls were D’ =0.38 and r2 = 0.02. After analysing measure of LD for tri-allelic 5-HTTLPR (rs25531)-STin2, values were D’ =0.34, r2 = 0.06 in CAD patients and D’ =0.31, r2 = 0.03 in controls.

**Table 4:** Haplotype analysis for 5-HTTLPR and STin2 polymorphisms of *SLC6A4* gene with CAD

Haplotypes	Frequency		OR [95% C.I.]	*p*-value
	CAD cases (*n* = 400)	Controls (*n* = 400)		
S-12	0.47	0.48	0.96 (0.79–1.16)	0.7
L-12	0.24	0.18	1.45 (1.14–1.85)	0.002*
S-10	0.24	0.29	0.77 (0.62–0.96)	0.02*
L-10	0.05	0.047	1.03 (0.62–1.62)	0.9

*Significant *p*-values

**Table 5:** Haplotype analysis for rs25531 and STin2 polymorphisms of *SLC6A4* gene with CAD

Haplotypes	Frequency		OR [95% C.I.]	*p*-value
	CAD cases (*n* = 400)	Controls (*n* = 400)		
S’-12	0.63	0.59	1.18 (0.97–1.45)	0.1
L_A_-12	0.08	0.07	1.15 (0.80–1.67)	0.4
S’-10	0.196	0.25	0.72 (0.57–0.91)	0.01*
L_A_-10	0.095	0.07	1.09 (0.78–1.54)	0.6

*Significant *p*-values

### Non-genetic risk factors

Various modifiable and non-modifiable non-genetic parameters were explored in the present study. Regarding age, the CAD patients were slightly older than the controls, with an average age of 56.42 years compared to 52.05 years in the control group (*p* < 0.0002). Both BMI and WHR were significantly higher in patients than in controls (*p* < 0.0001 and *p* = 0.008, respectively). Smoking behaviour emerged as a prominent risk factor associated with CAD in this study. The prevalence of smoking was higher among patients (39.75%) than among controls (12.5%). The proportions of current smokers, ex-smokers, and non-smokers in cases were 24.5%, 15.25%, and 60.25%, respectively, compared to 10.25%, 2.25%, and 87.5% in controls. Odds ratio analysis indicated that smoking contributed an approximately 4.6-fold increase in the risk of developing CVD within our population. The prevalence of family history for CVD/MI was 21.5% in patients versus 10% in controls, while for HTN, it was 30% and 14.5%, respectively, and for DM, 22.5% and 8.75%. (Table 6).

**Table 6:** Various non-genetic parameters and their association with CAD

S. No.	Parameters	CAD Patients (*N* = 400)	Controls (*N* = 400)	OR (95% C.I.)	*p*-value
1.	***Smoking*** Current Ex-smokers Non-smokers	159 (39.75%) 98 (24.5%) 61 (15.25%) 241 (60.25%)	50 (12.5%) 41 (10.25%) 9 (2.25%) 350 (87.5%)	**4.62**(3.23–6.60) Ref. (1)	< 0.0001* -
2.	***Eating habit*** Non-vegetarian Vegetarian	270 (67.5%) 130 (32.5%)	233 (58.25%) 167 (41.75%)	**1.49**(1.12–1.99) Ref. (1)	0.007* -
3.	***Fats intake*** Saturated Unsaturated Both	148 (37%) 136 (34%) 116 (29%)	121 (30.25%) 200 (50%) 79 (19.75%)	**1.80**(1.30–2.49) Ref. (1) **2.16**(1.51–3.09)	0.0004* - < 0.0001*
4.	***History of HTN*** Yes No	205 (51.25%) 194 (48.5%)	- -	- -	- -
5.	***History of DM*** Yes No	143 (35.75%) 257 (64.25%)	- -	- -	- -

## Discussion

Physiological implication of the *SLC6A4* gene in the cardiovascular system includes the proliferation of vascular smooth muscle cell, which is a part of the atherosclerosis process [24–26], vasoconstriction of the arterial wall, endothelial damage and pulmonary arterial hypertension [27, 28]. The majority of the available research has been done on bi-allelic 5-HTTLPR polymorphism. In contrast, data are scarce on the tri-allelic version, which is more informative in depicting genotype-phenotype correlation. To the best of our knowledge, the present study is the first in the Jammu region of the Jammu and Kashmir state of Indian population to evaluate the association of *SLC6A4* polymorphism with the manifestation of CAD. In view of 5-HTTLPR polymorphism, Asians tend to have the highest S-allele frequency [29], which is reported in the present investigation. The frequency of the S-allele reported in our study is similar to previously reported in other North Indian populations [30] but slightly higher than in the South Indian population [31]. In contrast, a higher frequency of L-allele was reported in individuals with European ancestry compared to the present study [32]. We found that the 5-HTTLPR L-carriers have an increased risk of developing CAD, and the LL-genotype showed a significant association with CAD. There are similar reports on association of LL-genotype with distinct acquired CVD phenotypes such as MI [33, 34], increased heart rate response to mental stress [35, 36], increased levels of LDL-C [37], CAD [7, 37], VSD related pulmonary arterial hypertension [38] and primary pulmonary hypertension [26]. The observed frequency of the S’ allele and LA allele in the present study is higher than the South Indian population [31]. In contrast, a similar frequency is observed in the Gujarati Indian in Houston, Texas population (1000 Genomes Phase 3). Williams and researchers have reported that the frequency of the S’ allele (rs25531) is relatively higher than the S allele (5-HTTLPR) in the Asian population [19], which is in line with the present study. These variations observed in allele frequencies suggested that population diversity plays a critical role in defining CAD susceptibility and probably accounts for the differences observed in the results of association studies.

Our study found that the bi-allelic 5-HTTLPR polymorphism is associated with CAD while combining SNP A to G (rs25531) in the 5-HTTLPR allele does not show any significant association with CAD risk. However, contradictory results were observed about higher systolic blood pressure and hypertension [19, 39]. Relying solely on bi-allelic 5-HTTLPR can overestimate the proportion of the high-activity of L-allele in a study population, leading to a potentially biased genotype-phenotype correlation. To address this issue, we also examined the tri-allelic 5-HTTLPR (rs25531) polymorphism and found no significant association with CAD risk in the North Indian population of Jammu. This finding confirmed that it is better to consider VNTR (5-HTTLPR) and SNP (rs25531) together when studying the association of *SLC6A4* to generate a true association rather than relying on biallelic 5-HTTLPR alone, which may produce false positive associations. Furthermore, we observed that the frequencies of STin2.12 and STin2.10 alleles are the same as reported earlier in the north Indian population [20] and South Indian population [21]. Unlike 5-HTTLPR, the VNTR STin2 has not been explicitly explored about its involvement in the manifestation of CAD and its phenotype. Our study was the first to evaluate the association of STin2 polymorphism with the susceptibility of CAD, and we observed a lack of association in the North Indian population of Jammu.

Our present study analyzed the haplotype combinations and LD patterns of *SLC6A4* variants to understand their association on susceptibility and disease progression of CAD. We found that L-12 and LA-10 haplotypes were associated with CAD. Additionally, all examined *SLC6A4* polymorphisms showed slight LD in both CAD patients and controls. This is the first report on haplotype analysis of *SLC6A4* polymorphisms and their potential association with the increased risk of CAD.

Concerning the non-genetic risk factors being examined, smoking has been identified as the principal risk factor for the development of CAD, as established by numerous studies both regionally and globally [40–42]. Moreover, the consumption of high-calorie foods and saturated fats, coupled with a lack of physical activity, has a direct impact on human health. Saturated fatty acids elevate total and low-density lipoprotein (LDL) cholesterol, thereby increasing the risk of cardiovascular diseases through a process known as atherosclerosis [43]. Limiting the intake of saturated fats and switching to unsaturated fatty acids (polyunsaturated fatty acids - PUFA) is a crucial step in managing dyslipidaemia and reducing cardiovascular complications in cardiac patients.

Thus, the present findings have highlighted the potential role of SLC6A4 haplotypes in CAD susceptibility and underscored the importance of lifestyle factors in disease progression. Given the complex interplay between genetic and environmental risk factors, further large-scale studies are needed to validate our findings.

## Conclusion

Our current case-control study, employing triallelic 5-HTTLPR genotypes and STin2, indicates that the SLC6A4 gene polymorphism is not associated with CAD susceptibility; however, smoking, as a non-genetic risk factor, is linked to increased risk CAD.

### Limitations of the study

One of the key limitations of the present study is its relatively small sample size, which may reduce the statistical power to detect weaker associations. Secondly, the study is geographically confined to the Jammu region of J&K state, which may limit the generalizability of the findings to other population groups with differing genetic backgrounds and environmental exposures. Additionally, the study concentrated only on a few polymorphisms within the SLC6A4 gene, and other genetic variants and gene-gene interactions were not explored. The study also relied on the self-reported health histories for the control selection, which may contribute toward selection bias.

## Data Availability

Original data and materials were obtained upon reasonable request from the corresponding author.

## Abbreviations

SLC6A4: Sodium dependent serotonin transporter
5-HTTLPR: Serotonin-transporter-linked promoter region
STin2: Serotonin transporter intronic enhancer
5-HTT: Serotonin transporter
CAD: Coronary artery disease
PCR: Polymerase chain reaction
CVD: Cardiovascular diseases

## References

[1] Khera AV, Kathiresan S. Genetics of coronary artery disease: discovery, biology and clinical translation. Nat Rev Genet. 2017;18(6):331–44. 28286336 10.1038/nrg.2016.160 PMC5935119

[2] Musunuru K, Kathiresan S. Genetics of common, complex coronary artery disease. Cell. 2019;177(1):132–45. 30901535 10.1016/j.cell.2019.02.015

[3] Fox KAA, Metra M, Morais J, Atar D. The myth of ‘stable’ coronary artery disease. Nat Rev Cardiol. 2020;17(1):9–21. 31358978 10.1038/s41569-019-0233-y

[4] Malakar AK, Choudhury D, Halder B, Paul P, Uddin A, Chakraborty S. A review on coronary artery disease, its risk factors, and therapeutics. J Cell Physiol. 2019;234(10):16812–23. 30790284 10.1002/jcp.28350

[5] Okaty BW, Commons KG, Dymecki SM. Embracing diversity in the 5-HT neuronal system. Nat Rev Neurosci. 2019;20(7):397–424. 30948838 10.1038/s41583-019-0151-3

[6] Coleman JA, Yang D, Zhao Z, Wen PC, Yoshioka C, Tajkhorshid E, et al. Serotonin transporter-ibogaine complexes illuminate mechanisms of Inhibition and transport. Nature. 2019;569(7754):141–45. 31019304 10.1038/s41586-019-1135-1 PMC6750207

[7] Bozzini S, Gambelli P, Boiocchi C, Schirinzi S, Falcone R, Buzzi P, et al. Possible role of brain-derived neurotrophic factor and serotonin transporter gene polymorphisms. Int J Mol Med. 2009;24(6):813–18. 19885623 10.3892/ijmm_00000297

[8] Ni W, Watts SW. 5-Hydroxytryptamine in the cardiovascular system: focus on the serotonin transporter (SERT). Clin Exp Pharmacol Physiol. 2006;33(7):575–83. 16789923 10.1111/j.1440-1681.2006.04410.x

[9] Lesch KP, Balling U, Gross J, Strauss K, Wolozin BL, Murphy DL, et al. Organization of the human serotonin transporter gene. J Neural Transm Gen Sect. 1994;95(2):157–62. 7865169 10.1007/BF01276434

[10] Santangelo AM, Ito M, Shiba Y, Clarke HF, Schut EH, Cockcroft G, et al. Novel primate model of serotonin transporter genetic polymorphisms associated with gene expression, anxiety and sensitivity to antidepressants. Neuropsychopharmacology. 2016;41(9):2366–76. 26997299 10.1038/npp.2016.41 PMC4946067

[11] Botton MR, Yang Y, Scott ER, Desnick RJ, Scott SA. Phased haplotype resolution of the SLC6A4 promoter using Long-Read single molecule Real-Time (SMRT) sequencing. Genes (Basel). 2020;11(11):1333. 10.3390/genes11111333 PMC7696006 33198140

[12] Nakamura M, Ueno S, Sano A, Tanabe H. The human serotonin transporter gene linked polymorphism (5-HTTLPR) shows ten novel allelic variants. Mol Psychiatry. 2000;5(1):32–8. 10673766 10.1038/sj.mp.4000698

[13] Houwing DJ, Buwalda B, van der Zee EA, de Boer SF, Olivier JDA. The serotonin transporter and early life stress: translational perspectives. Front Cell Neurosci. 2017;11:117. 28491024 10.3389/fncel.2017.00117 PMC5405142

[14] Fratelli C, Siqueira J, Silva C, Ferreira E, Silva I. 5HTTLPR genetic variant and major depressive disorder: A review. Genes (Basel). 2020;11(11):1260. 33114535 10.3390/genes11111260 PMC7692865

[15] Chang CC, Chang HA, Fang WH, Chang TC, Huang SY. Gender-specific association between serotonin transporter polymorphisms (5-HTTLPR and rs25531) and neuroticism, anxiety and depression in well-defined healthy Han Chinese. J Affect Disord. 2017;207:422–28. 27788383 10.1016/j.jad.2016.08.055

[16] Kalungi A, Womersley JS, Kinyanda E, Joloba ML, Ssembajjwe W, Nsubuga RN, et al. The 5-HTTLPR-rs25531 S-A-S-A haplotype and chronic stress moderate the association between acute stress and internalizing mental disorders among HIV + Children and adolescents in Uganda. Front Genet. 2021;12:649055. 33968131 10.3389/fgene.2021.649055 PMC8104030

[17] Kuhn L, Noack H, Skoluda N, Wagels L, Röhr AK, Schulte C, et al. The association of the 5-HTTLPR polymorphism and the response to different stressors in healthy males. J Neural Transm (Vienna). 2021;28(9):1347–59. 10.1007/s00702-021-02390-4 PMC8423678 34374855

[18] Hu XZ, Lipsky RH, Zhu G, Akhtar LA, Taubman J, Greenberg BD. Serotonin transporter promoter Gain-of-Function genotypes are linked to Obsessive-Compulsive disorder. Am J Hum Genet. 2006;78(5):815–26. 16642437 10.1086/503850 PMC1474042

[19] Williams RB, Bishop GD, Haberstick BC, Smolen A, Brummett BH, Siegler IC, et al. Population differences in associations of serotonin transporter promoter polymorphism (5HTTLPR) di- and triallelic genotypes with blood pressure and hypertension prevalence. Am Heart J. 2017;185:110–22. 28267464 10.1016/j.ahj.2016.12.013 PMC5473420

[20] Joshi G, Pradhan S, Mittal B. No direct association of serotonin transporter (STin2 VNTR) and receptor (HT 102T > C) gene variants in genetic susceptibility to migraine. Dis Markers. 2010;29(5):223–29. 21206007 10.3233/DMA-2010-0752 PMC3835529

[21] Vijayan NN, Iwayama Y, Koshy LV, Natarajan C, Nair C, Allencherry PM. Evidence of association of serotonin transporter gene polymorphisms with schizophrenia in a South Indian population. J Hum Genet. 2009;54(9):538–42. 19713975 10.1038/jhg.2009.76

[22] Sambrook J, Russell DW, Molecular Cloning. A Laboratory Manual, 2nd edition. 2001. Cold Spring Harbour Laboratory Press, Cold Spring Harbor.

[23] Shen J, Li Z, Chen J, Song Z, Zhou Z, Shi Y. SHEsisPlus, a toolset for genetic studies on polyploid species. Sci Rep. 2016;6:24095. 27048905 10.1038/srep24095 PMC4822172

[24] Crowley ST, Dempsey EC, Horwitz KB, Horwitz LD. Platelet induced vascular smooth muscle cell proliferation is modulated by the growth amplification factors: serotonin and adenosine diphosphate. Circulation. 1994;90(4):1908–18. 7923679 10.1161/01.cir.90.4.1908

[25] Sauls K, de Vlaming A, Harris BS, Williams K, Wessels A, Levine RA, et al. Developmental basis for filamin-A-associated myxomatous mitral valve disease. Cardiovasc Res. 2012;96(1):109–19. 22843703 10.1093/cvr/cvs238 PMC3444235

[26] Moyer AM, Walker DL, Avula R, Lapid MI, Kung S, Bryant SC, et al. Relationship of genetic variation in the serotonin transporter gene (SLC6A4) and congenital and acquired cardiovascular diseases. Genet Test Mol Biomarkers. 2015;19(3):115–23. 25671637 10.1089/gtmb.2014.0250

[27] Golino P, Piscione F, Willerson JT, Cappelli-Bigazzi M, Focaccio A, Villari B, et al. Divergent effects of serotonin on coronary-artery dimensions and blood flow in patients with coronary atherosclerosis and control patients. N Engl J Med. 1991;324(10):641–48. 1994246 10.1056/NEJM199103073241001

[28] Zhang LJ, Zeng XT, Zhao MJ, He DF, Liu JY, Liu MY. The important effect of 5-HTTLPR polymorphism on the risk of depression in patients with coronary heart disease: a meta-analysis. BMC Cardiovasc Disord. 2020;20(1):141. 32188408 10.1186/s12872-020-01424-1 PMC7081537

[29] Kangelaris KN, Vittinghoff E, Otte C, Na B, Auerbach AD, Whooley MA. Association between a serotonin transporter gene variant and hopelessness among men in the heart and soul study. J Gen Intern Med. 2010;25(10):1030–37. 20509052 10.1007/s11606-010-1403-0 PMC2955461

[30] Basu A, Chadda RK, Sood M, Kaur H, Kukreti R. Association of serotonin transporter (SLC6A4) & receptor (5HTR1A, 5HTR2A) polymorphisms with response to treatment with Escitalopram in patients with major depressive disorder: A preliminary study. Indian J Med Res. 2015;142(1):40–5. 26261165 10.4103/0971-5916.162094 PMC4557249

[31] Tibrewal P, Kumar HBK, Shubha GN, Subhashree D, Purushottam M, Thennarasu K, et al. Association of serotonin transporter gene polymorphisms with obsessive-compulsive disorder (OCD) in a South Indian population. Indian J Med Res. 2010;132(6):690–95. 21245616 PMC3102456

[32] Machado RD, Koehler R, Glissmeyer E, Veal C, Suntharalingam J, Kim M, et al. Genetic association of the serotonin transporter in pulmonary arterial hypertension. Am J Respir Crit Care Med. 2006;173(7):793–97. 16399993 10.1164/rccm.200509-1365OC

[33] Fumeron F, Betoulle D, Nicaud V, Evans A, Kee F, Ruidavets JB, et al. Serotonin transporter gene polymorphism and myocardial infarction: etude Cas-Témoins de l’infarctus du myocarde (ECTIM). Circulation. 2002;105(25):2943–45. 12081984 10.1161/01.cir.0000022603.92986.99

[34] Coto E, Reguero JR, Alvarez V, Morales B, Batalla A, González P, et al. 5Hydroxytryptamine 5-HT2A receptor and 5-hydroxytryptamine transporter polymorphisms in acute myocardial infarction. Clin Sci (Lond). 2003;104(3):241–5. 12605580 10.1042/CS20020246

[35] Williams RB, Marchuk DA, Gadde KM, Barefoot JC, Grichnik K, Helms MJ, et al. Central nervous system serotonin function and cardiovascular responses to stress. Psychosom Med. 2001;63(2):300–5. 11292279 10.1097/00006842-200103000-00016

[36] Porcelli S, Fabbri C, Serretti A. Meta-analysis of serotonin transporter gene promoter polymorphism (5-HTTLPR) association with antidepressant efficacy. Eur Neuropsychopharmacol. 2012;22(4):239–58. 22137564 10.1016/j.euroneuro.2011.10.003

[37] Fischer P, Gruenblatt E, Pietschmann P, Tragl KH. Serotonin transporter polymorphism and LDL-cholesterol. Mol Psychiatry. 2006;11(8):707–9. 16868569 10.1038/sj.mp.4001837

[38] Cao H, Gu H, Qiu W, Zuo W, Zheng L, Wang Z, et al. Association study of serotonin transporter gene polymorphisms and ventricular septal defects related possible pulmonary arterial hypertension in Chinese population. Clin Exp Hypertens. 2009;31(7):605–14. 19886858 10.3109/10641960902993061

[39] Brummett BH, Siegler IC, Ashley-Koch A, Williams RB. Effects of 5HTTLPR on cardiovascular response to an emotional stressor. Psychosom Med. 2011;73(4):318–22. 21364197 10.1097/PSY.0b013e3182118c16 PMC3090460

[40] Raina JK, Sharma M, Sethi S, Panjaliya RK, Bakaya A, Kumar P. A pilot study on recognition and prevalence of risk factors for cardiovascular diseases in North Indian populace of Jammu and Kashmir. J Hum Ecol. 2018;62(1–3):47–57.

[41] Kalra S, Narain S, Karki P, Ansari JA, Ranabhat K, Basnet N. Prevalence of risk factors for coronary artery disease in the community in Eastern Nepal- a pilot study. J Assoc Physicians India. 2011;59:1–2. 21751607

[42] Sekhri T, Kanwar RS, Wilfred R, Chugh P, Chhillar M, Aggarwal R, et al. Prevalence of risk factors for coronary artery disease in an urban Indian population. BMJ Open. 2014;4:e005346. 25488095 10.1136/bmjopen-2014-005346 PMC4281543

[43] Hansson GK. (2005). Inflammation, Atherosclerosis, and Coronary Artery Disease. N Engl J Med. 2005; 352: 1685–1695. 10.1056/NEJMra043430 15843671

